# Nutritional Support for Pediatric Gastroenterology Patients

**DOI:** 10.3390/nu17121974

**Published:** 2025-06-11

**Authors:** Víctor Manuel Navas-López, Marta Herrador-López, Rafael Martín-Masot

**Affiliations:** Pediatric Gastroenterology and Nutrition Unit, Hospital Regional Universitario de Malaga, 29010 Málaga, Spain; herradorlopezm@gmail.com (M.H.-L.); rafammgr@gmail.com (R.M.-M.)

Understanding and addressing nutritional challenges related to cow’s milk consumption during infancy and childhood remains a critical and evolving field within pediatric nutrition and gastroenterology. The early introduction of cow’s milk protein into the infant diet, coupled with the widespread prevalence of cow’s milk-related disorders, has prompted extensive research into both immune-mediated reactions, such as cow’s milk allergy (CMPA) [1], and non-immune disorders like lactose intolerance. This Special Issue brings together five valuable contributions that not only reflect recent scientific advancements but also help clarify some of the longstanding diagnostic and therapeutic uncertainties that continue to affect clinical decision-making. Importantly, these studies also provide a clear direction for future research priorities that may ultimately lead to improved outcomes for pediatric patients.

One of the main challenges in pediatric practice is the frequent confusion between CMPA and lactose intolerance, especially in infants and young children, due to overlapping gastrointestinal symptoms [1]. This diagnostic ambiguity often results in delayed diagnosis, inappropriate interventions, and, in some cases, unnecessary elimination diets that can jeopardize nutritional status during a critical period of growth [2]. Two reviews within this issue tackle this clinical dilemma from complementary perspectives. The first provides a detailed clinical and mechanistic comparison between lactose intolerance and CMPA, emphasizing the need for improved diagnostic criteria and professional education to reduce misclassification and its consequences (contribution 1) [3,4]. The second review explores the potential of prebiotic strategies, such as the administration of galactooligosaccharides (GOS), to mitigate lactose intolerance symptoms by modifying the composition and function of gut microbiota (contribution 2). Although the evidence is still preliminary, this approach represents a promising avenue that could lead to personalized nutritional therapies for individuals with lactase non-persistence, particularly in populations where dairy remains a key nutritional component [5,6,7,8,9].

Biomarker development for non-IgE-mediated CMPA, and specifically cow’s milk-protein-induced allergic proctocolitis, is another emerging area of interest. Currently, the diagnosis of cow’s milk-protein-induced allergic proctocolitis relies heavily on clinical observation and food challenge tests, which are often invasive, time-consuming, and stressful for both infants and families [1]. In this context, a prospective study examining fecal calprotectin (FC) [10] and zonulin-related proteins [11] in infants with cow’s milk-protein-induced allergic proctocolitis offers significant insights (contribution 3). The findings demonstrate that a milk-free diet leads to a marked reduction in both biomarkers, suggesting their potential utility for monitoring disease resolution. However, the high inter-individual variability of FC and the absence of zonulin-related protein secretion in a subset of patients limit their diagnostic precision. These results highlight the urgent need for further studies to validate non-invasive markers in larger, more heterogeneous pediatric populations and to explore their role not only in diagnosis, but also in predicting disease course and tolerance acquisition.

Beyond allergy and intolerance, nutritional screening and assessment in children with complex medical conditions remains a critical yet often under-addressed area. Disease-related malnutrition (DRM) continues to affect a substantial proportion of hospitalized pediatric patients, with consequences that include impaired immune function, delayed recovery, and increased healthcare utilization [12,13]. The systematic review of nutritional screening tools presented in this Special Issue systematically evaluates existing instruments, identifying the Pediatric Yorkhill Malnutrition Score (PYMS) as having the highest diagnostic yield (contribution 4). However, the review also reveals a considerable degree of methodological heterogeneity and a lack of universally accepted reference standards, which complicates comparison across studies. This underscores the need to establish consensus on the essential components of pediatric nutritional screening tools and to conduct longitudinal validation studies across diverse healthcare settings. The integration of such tools into routine pediatric care could support earlier identification of at-risk children and more timely intervention.

Pediatric dysphagia represents another major nutritional risk factor that is often overlooked or insufficiently managed. Children with oropharyngeal dysphagia, particularly those with neurological impairments, face a heightened risk of malnutrition due to reduced oral intake, increased energy requirements, and frequent feeding difficulties [14]. The quasi-experimental study included in this Special Issue assessed the impact of an individualized nutritional and rehabilitative approach in a cohort of children with dysphagia (contribution 5). The findings are encouraging, with significant improvements observed in caloric intake and food texture tolerance following intervention, highlighting the effectiveness of multidisciplinary care strategies. This study exemplifies the importance of integrating speech therapy, nutritional assessment, and caregiver education into a comprehensive management plan for children with dysphagia. Future research should aim to evaluate the long-term outcomes of such interventions, their cost-effectiveness, and their impact on quality of life for both children and their families.

Taken together, the contributions to this Special Issue uncover important knowledge gaps and provide a roadmap for future research. There is a clear need for better diagnostic markers for non-IgE-mediated food allergies, more accurate and feasible nutritional screening tools, and effective, evidence-based dietary strategies to manage both lactose intolerance and dysphagia. Furthermore, cross-disciplinary collaboration—among pediatricians, gastroenterologists, nutritionists, speech therapists, and researchers—will be essential to translating these findings into clinical practice.

We extend our deepest gratitude to all the authors, reviewers, and editors who contributed to the development of this Special Issue. Their collective efforts not only advance our understanding of pediatric nutritional disorders but also provide practical insights that may improve clinical care. It is our hope that the findings presented here will inspire further innovation, foster interdisciplinary collaboration, and ultimately contribute to better health outcomes for infants and children worldwide.
